# Diagnostic Accuracy of Dual-Energy CT Parameters for Discrimination of Hypodense Liver Lesions in Patients Affected by Colorectal Cancer

**DOI:** 10.3390/jcm14175929

**Published:** 2025-08-22

**Authors:** Tommaso D’Angelo, Ludovica R. M. Lanzafame, Timo Steinert, Silvio Mazziotti, Manuela França, Ahmed E. Othman, Mirela Dimitrova, Scherwin Mahmoudi, Ibrahim Yel, Leona S. Alizadeh, Leon D. Grünewald, Vitali Koch, Simon S. Martin, Thomas J. Vogl, Christian Booz

**Affiliations:** 1 Diagnostic and Interventional Radiology Unit, BIOMORF Department, University Hospital Messina, 98124 Messina, Italy; 2 Division of Experimental Imaging, Department of Diagnostic and Interventional Radiology, University Hospital Frankfurt, 60590 Frankfurt am Main, Germany; 3 Department of Diagnostic and Interventional Radiology, University Hospital Frankfurt, 60590 Frankfurt am Main, Germany; 4 Radiology Department, Centro Hospitalar Universitário do Porto, University of Porto, 4050-342 Porto, Portugal; 5 Institute of Neuroradiology, Johannes Gutenberg University Hospital Mainz, 55131 Mainz, Germany

**Keywords:** dual energy computed tomography, colorectal cancer, neoplasm metastasis, cysts

## Abstract

**Objective**: The aim of this study was to evaluate the diagnostic accuracy of conventional CT values and the dual-energy computed tomography (DECT)-derived effective atomic number (Zeff), fat fraction (FF) and iodine concentration (IC) for the differentiation of hypodense liver lesions in patients with colorectal cancer (CRC). **Methods**: One hundred and twenty patients (mean age: 65 ± 12 years) affected by CRC who underwent dual-source DECT as part of tumor staging between December 2015 and June 2023 were retrospectively evaluated. Spectral datasets were reconstructed for each patient and regions of interest were applied at the level of hypodense liver lesions to collect CT, Zeff, FF and IC values. To assess diagnostic accuracy, receiver operating characteristic (ROC) curves were constructed to evaluate the area under the curve (AUC), sensitivity, and specificity using biopsy or MRI (in cases when biopsy was not indicated or feasible) as a reference standard. The Youden index was used to identify optimal cut-off values for potential clinical applications. **Results**: A total of 223 lesions (147 metastases and 76 cysts) were evaluated. CT, FF and IC values differed significantly between metastases and cysts (*p* < 0.0001), showing high diagnostic accuracy. FF showed significantly higher diagnostic accuracy compared to all other parameters (all *p* ≤ 0.0074), with an AUC value of 0.97 (95% CI: 0.94–0.99). For a cut-off > 15.9, the sensitivity reached 91.8% (95% CI: 86.2–95.7) and the specificity reached 98.7% (95% CI: 92.9–100). Zeff numbers did not differ considerably (*p* = 0.781) between the two entities and demonstrated a lower AUC (0.511; 95% CI: 0.44–0.58). **Conclusions**: FF measurements proved to have high diagnostic accuracy compared to CT values, IC, and Zeff in the evaluation of hypodense liver lesions in patients suffering from colorectal carcinoma.

## 1. Introduction

Colorectal cancer (CRC) is one of the leading global causes of cancer-related mortality, ranking third among cancers in males and second in females [1,2]. Despite advances in early detection and treatment, the incidence of CRC continues to rise, driven by genetic predispositions, environmental factors, and lifestyle changes [3,4,5].

Approximately 25–30% of patients with CRC develop liver metastases during their disease [6]. The early recognition of metastatic disease is imperative to defining proper patient management, allowing the evaluation of the most appropriate therapeutic approach and prolonging patient survival [7,8,9]. Contrast-enhanced computed tomography (CT) is usually used for the staging and follow-up in oncologic patients due to its widespread availability, short acquisition times, and high spatial resolution, which allow the definition of the invasion to surrounding structures and lymphatic, hematogenous and peritoneal spread. According to international society guidelines, contrast-enhanced CT is considered the modality of choice for the detection of CRC liver metastases. Nonetheless, the diagnosis of hypodense liver lesions often represents a challenge as cysts and metastases can have similar CT values due to low vascularity and the presence necrotic processes, especially in case of small-sized lesions which are considered “too small to characterize”. Therefore, magnetic resonance (MRI) is frequently performed as a further diagnostic test due to its high diagnostic value in characterizing liver lesions [10,11,12]. However, the limited availability of MRI often leads to a delay in the diagnosis, and its execution is associated with significant contraindications.

Dual-energy CT (DECT) has emerged as a promising advancement in oncologic imaging, offering the ability to differentiate tissue composition using material decomposition algorithms and energy-dependent attenuation profiles. DECT-derived algorithms have demonstrated a significant impact on daily clinical practice. For instance, virtual monoenergetic images (VMI) enhance the detection of small hypoattenuating liver lesions in patients with extrahepatic malignancies. Additionally, iodine maps derived through three-material decomposition enable the quantification and visualization of iodine distribution, improving characterization of hypo- and hypervascular lesions and offering insight into tumor vascularity. The fat fraction (FF) algorithm allows for the non-invasive quantification of hepatic steatosis by generating fat maps expressed as volume percentages, which have shown good correlation with MR spectroscopy findings. Lastly, Z-effective maps provide estimates of the average atomic composition of tissues and may offer further characterization potential [13,14,15,16,17,18,19,20].

However, to date, no comparative study has directly assessed the diagnostic performance of these models in evaluating hypodense hepatic lesions in patients with colorectal cancer.

This study aimed to evaluate and compare the diagnostic performance of CT values and quantitative DECT-derived parameters for the differentiation of hypodense liver lesions in patients affected by CRC using MRI or biopsy as a reference standard and to determine appropriate threshold values for clinical application.

## 2. Materials and Methods

The institutional review board of Goethe University Frankfurt approved this retrospective study (Protocol No. 2023-1216). The requirement to obtain written informed consent was waived.

### 2.1. Study Population

**Figure 1:**
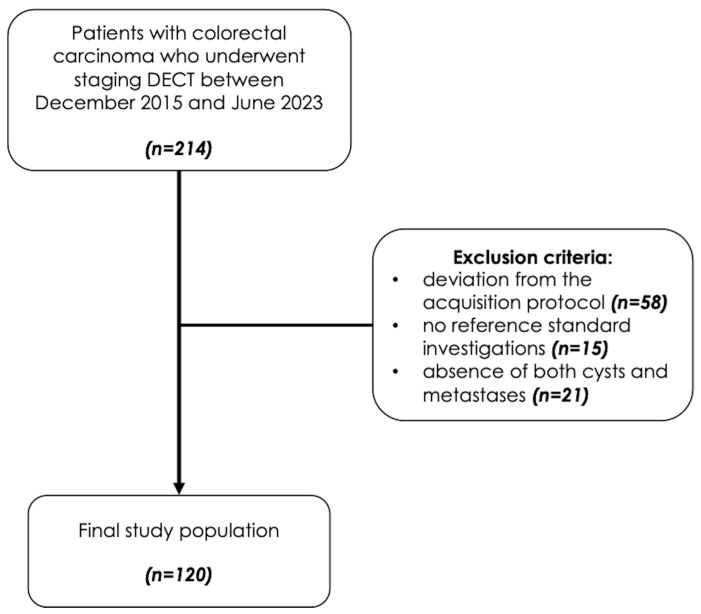
Study flowchart.

Two hundred and fourteen consecutive patients with histologically confirmed colorectal cancer who underwent routine third-generation dual-source DECT staging between December 2015 and June 2023 were retrospectively evaluated. The exclusion criteria included deviation from the acquisition protocol (*n* = 58), no reference standard investigations (*n* = 15), and the absence of both cysts and metastases (*n* = 21). The final study population consisted of 120 patients. Figure 1 illustrates the patient selection process.

### 2.2. DECT Scan Protocol

Chest-abdominal staging CT examinations were acquired using a third-generation dual-source dual-energy CT system (Somatom Force, Siemens Healthineers, Forchheim, Germany). Intravenous contrast medium (Iomeron 400 mgI/mL, Bracco, Milan, Italy) was administered at a dose of 1.3 mL/kg and an injection rate of 3 mL/s via a superficial forearm vein, following a fixed delay of 85 s. All scans were acquired with patients in the supine position, employing a craniocaudal scan direction and dual-energy acquisition mode, wherein the two X-ray tubes operated simultaneously at different tube voltages (tube A: 100 kV and tube B: Sn150 kV with a tin filter). The rotation time was 0.5 s, the collimation width was 128 × 0.6 mm, and the pitch was 0.6 mm.

### 2.3. CT Image Reconstruction

In each CT scan, three different image sets were acquired, namely 100 kV, Sn150 kV, and the calculated weighted average (ratio 0.5:0.5) to resemble the image properties of a single-energy 120 kVp scan. Standard reconstructions (axial, coronal, and sagittal: section thickness, 1 mm; increment, 0.75 mm) were generated with a dual-energy medium-soft convolution kernel (Qr40, advanced model-based iterative reconstruction [ADMIRE] level of 3) for the high- and low-kilovolt series. All reconstructions were transferred to the picture archiving and communication system (PACS) for image evaluation.

### 2.4. Measurements of CT, FF, Zeff and IC Values

DECT image series was post-processed on a dedicated DECT workstation (syngo.via, version VB10B, Siemens Healthineers, Forchheim, Germany) using the default iodine subtraction algorithm (Liver VNC, Siemens Healthineers, Forchheim, Germany) based on a three-material decomposition (iodine, fat, and tissue) to calculate quantitative CT data from portal venous-phase images, including iodine concentration (IC) and fat fraction (FF) measurements. The Rho/Z maps algorithm was used to achieve tissue differentiation based on the effective atomic number (Zeff). All images from the spectral algorithms were reconstructed using the same slice thickness and increment parameters as observed for the conventional series.

**Figure 2:**
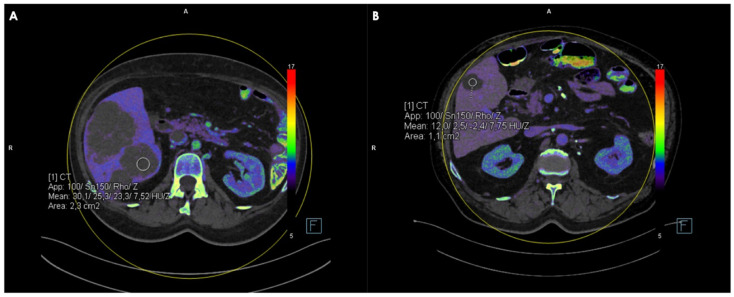
Axial portal-venous phase Z-effective maps showing ROI positioning in a metastatic liver lesion (**A**) and in a benign cyst (**B**).

Manually delineated circular regions of interest (ROIs), with the largest possible diameter and minimum and maximum area values ranging between 0.4 and 4 cm^2^, were placed by two radiologists with 5 and 12 years of experience in oncologic imaging on axial conventional and spectral reconstructions of hypodense liver lesions to obtain FF, IC, Zeff, and CT values, avoiding lesion margins, large blood vessels, and surrounding artifacts (Figure 2). Each measurement was performed twice by each reader, recorded, and averaged. To reduce the risk of bias, the reader was blinded to patient information, medical history, and the type of reference standard used. Additionally, the images were evaluated in a randomized manner.

### 2.5. Reference Standard

MRI or biopsy served as a standard reference in this study for lesion definition. Biopsy was considered the gold standard when available (and thus when clinically indicated or feasible), while MRI was used in the remaining cases. Contrast-enhanced MRI examinations were acquired on a 3T platform (MAGNETOM Prisma FIT, Siemens Healthineers, Forchheim, Germany). The scan protocol included the following sequences: T2-weighted half-Fourier acquisition single shot turbo spin echo (HASTE) sequences in axial and coronal view, axial T1-weigthed gradient echo (GRE) in opposed-phase sequences, diffusion-weighted images in axial orientation, and axial dynamic contrast-enhanced T1-weighted sequences with fat saturation (VIBE). Unenhanced, arterial phase, portal-venous phase, and equilibrium phase images were acquired for dynamic liver examination. Gadobutrol (Gadovist, Bayer Healthcare, Leverkusen, Germany) at a dose of 0.01 mg/kg was administered through a superficial vein in the forearm at a flow rate of 2 mL/s followed by a 20 mL chaser of saline solution. The MRI results were interpreted by the same radiologists in consensus.

### 2.6. Statistical Analysis

Statistical analysis was performed using commercially available software (MedCalc Software Ltd., version 20, Ostend, Belgium). The Shapiro–Wilk test was used to analyze the normality of data. Numeric values of continuous variables were given as the mean ± SD if normally distributed, or as the median [IQR] for non-normally distributed data, and categoric variables were expressed as numbers or percentages. Normally distributed data were compared using a two-tailed *t*-test, whereas in cases in which the normality was uncertain, data were assessed using the Mann–Whitney test. The threshold for assessing statistical significance was set at *p* < 0.05. Intra- and inter-rater agreement between the measurements recorded by the two observers were assessed using the intraclass correlation coefficient (ICC). ICC values were interpreted as follows: poor reliability: <0.5; moderate reliability: 0.5–0.75; good reliability: 0.75–0.9; excellent reliability: >0.90. For the quantitative image analysis, receiver-operating characteristic (ROC) curve analysis was performed, the area under the curve (AUC) was calculated, and optimal cut-off values for the differentiation of liver lesions were determined by the Youden index. For these optimal cut-off values, sensitivity and specificity values were calculated.

## 3. Results

A total of 223 hypodense liver lesions (147 metastasis; 65.9% and 76 cysts; 34.1%) were evaluated in a population of 120 patients with confirmed colorectal cancer (mean age: 65 ± 12 years), consisting of 49 women (40.8%) and 71 men (59.2%). Of the total lesions, 145 (65.0%) were confirmed using contrast-enhanced MRI and 78 (35.0%) by histopathological biopsy. The mean lesion size was 2.2 ± 0.5 cm. Demographic characteristics are summarized in Table 1.

Liver metastases and cysts showed averaged CT values of 34.3 [27.8–42.5] HU and 11.9 [9.4–16.2] HU. FF and IC values were 25.3 [20.7–27.6] % and 1.6 [1.3–1.8] mg/mL for metastases and 7.2 [4.7–10.9] % and 0.7 [0.5–1] mg/mL for cysts, respectively.

**Figure 3:**
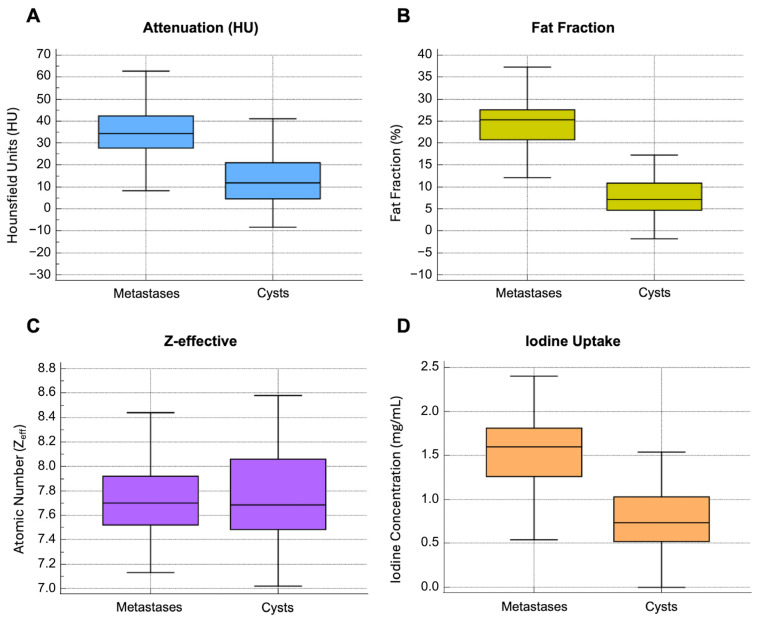
Box-and-whisker plots showing considerable differences in CT value, FF, and IC distribution in liver CRC metastases and benign cysts (*p* < 0.0001) (**A**,**B**,**D**). The Zeff values were comparable between the two lesion types (*p* = 0.781) (**C**).

CT, FF, and IC values showed a significant difference between benign liver cysts and CRC metastases (*p* < 0.0001). In contrast, Zeff did not differ significantly between the two categories of lesions (*p* = 0.781), with Zeff values of 7.7 [7.6–7.8] and 7.7 [7.5–8.1] for metastases and cysts, respectively. The results are summarized in Table 2, while Figure 3 shows the distribution of CT, FF, Zeff, and IC.

The ICC analysis demonstrated good intra- and inter-observer agreement, with values ranging from 0.85 to 0.94. The full results are reported in Table 3.

**Figure 4:**
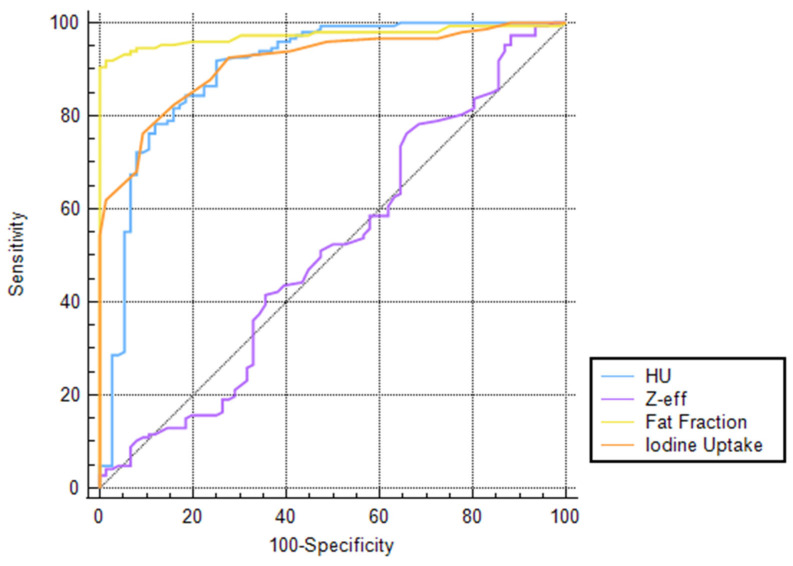
ROC curve comparison of CT, FF, Zeff, and IC values’ diagnostic accuracy evaluated using liver MRI or biopsy as a reference standard.

The ROC curves demonstrated that FF displays higher diagnostic accuracy for differentiating liver metastases and cysts compared to other parameters, with an AUC value of 0.97 (95% CI: 0.94–0.99), reaching, for a cut-off > 15.9, a sensitivity of 91.8% (95% CI: 86.2–95.7) and a specificity of 98.7% (95% CI: 92.9–100) and significantly outperforming all other parameters (all *p* ≤ 0.0074). On the other hand, Zeff values showed the lowest diagnostic performance, reaching an AUC value of 0.511 (95% CI: 0.44–0.58), with a sensitivity and specificity of 76.2% (95% CI: 68.5–82.8) and 34.2% (95% CI: 23.7–40), respectively, for a cut-off > 7.5. The complete results regarding the diagnostic accuracy of each reconstruction are detailed in Table 4. The diagnostic performance of CT values, FF, IC, and Zeff in differentiating liver lesions is displayed as ROC curves in Figure 4.

## 4. Discussion

This study is the first to evaluate and compare the diagnostic accuracy of conventional CT attenuation values and DECT-derived quantitative parameters in the differentiation of hypodense liver lesions in CRC patients. FF values showed substantially higher diagnostic performance compared to CT values, IC, and Zeff for the discrimination of liver metastases and cysts, with an AUC value of 0.97 (95% CI: 0.94–0.99). Other studies have demonstrated the utility of fat fraction (FF) in the diagnosis of various lesions. Winkelmann et al. identified the fat fraction as the most accurate parameter for the differential diagnosis between hamartomas and lung tumors, achieving an AUC of 0.96 (95% CI: 0.86–0.99) [21].

By contrast, Zeff maps failed to provide clinically meaningful differentiation, with an AUC of 0.511 and significantly lower sensitivity and specificity. These results highlight the limited utility of Zeff maps in this context, despite their theoretical potential. Previous studies have shown promise for Zeff in differentiating other lesion types, such as renal masses, where the atomic number offers clear compositional distinctions. Mileto et al. evaluated DECT atomic number maps’ performance in 206 patients for the discrimination of non-enhancing renal cysts and enhancing masses. The authors found significant difference in Zeff values between the two entities with elevated diagnostic accuracy (0.92; 95% CI: 0.89–0.94) when considering a threshold of 8.36, highlighting the ability of the atomic number in distinguishing renal cysts from enhancing masses [19]. This discrepancy underscores the need for further research into the optimization of Zeff reconstruction algorithms and their potential applications.

Although differences in acquisition, reconstruction, and post-processing techniques may have impacted the final appearance and diagnostic utility of the spectral Z maps, we believe that the observed discrepancies are likely multifactorial. In particular, lesion-specific histological features, such as a greater tendency toward necrotic or liquefactive degeneration in certain metastases, could lead to increased intralesional water content, potentially reducing the contrast between benign and malignant lesions on Zeff maps, while hemorrhagic or proteinaceous content within cystic lesions might have influenced the values in the opposite direction. However, findings from a separate study conducted at our institution demonstrated that atomic-number-based maps provided the highest diagnostic accuracy for differentiating melanoma metastases from benign cysts (AUC = 0.992; 95% CI: 0.956–1), supporting the value of this parameter in lesion characterization [22]. These conflicting results suggest that both technical factors and lesion biology may influence the performance of spectral imaging tools and should be considered when interpreting Z map findings.

Clinically, these findings hold relevant implications. The ability to accurately characterize hypodense liver lesions using a single contrast-enhanced CT examination—particularly through the integration of DECT-derived parameters—may reduce the need for additional imaging, such as MRI. This is especially valuable in the context of colorectal cancer staging and surveillance, where timely diagnosis is essential. In particular, the availability of fat fraction quantification directly from contrast-enhanced CT may streamline workflows, minimize diagnostic delays, and optimize resource utilization in routine oncologic care. CT plays a crucial role in staging disease and in the early recognition of lesions to identify patients eligible for surgical treatment. Patients eligible for surgical treatment should be assessed case-by-case on the basis of prognostic criteria. Currently, the resectability criteria no longer include the number, maximal size, and distribution of liver lesions; however, the degree of liver involvement affects the functional reserve after therapy. CT allows the evaluation of the liver functional reserve, the prediction of post-hepatectomy liver failure, and the identification of the presence and extension of extrahepatic disease, which are considered among the main factors that affect survival after therapy [23,24,25].

However, challenges persist. Hypovascular metastases, particularly those under 1 cm in size or located subcapsularly, may still evade characterization due to partial volume effects and reduced contrast-to-noise ratios [26,27,28]. These limitations necessitate continued innovation in CT acquisition and post-processing techniques, including thinner slice reconstructions and advanced noise reduction algorithms, to enhance diagnostic accuracy further [29].

An important aspect for future consideration is the diagnostic value of the evaluated CT parameters in true non-contrast (TNC) and virtual non-contrast (VNC) imaging in characterizing hypodense liver lesions. TNC imaging has long been a cornerstone for assessing baseline attenuation values, particularly for detecting calcifications or fat content, but it entails additional radiation exposure and lengthens imaging protocols. DECT-derived VNC imaging, on the other hand, offers the potential to approximate unenhanced images using contrast-enhanced data, thereby reducing patient radiation dose and streamlining workflows [30]. While VNC imaging has been successfully applied in various clinical scenarios, including renal lesion evaluation and the differentiation of adrenal adenomas, its application in the context of liver lesion characterization remains underexplored [31,32]. Preliminary studies suggest that VNC imaging might reliably approximate true attenuation values for liver lesions, but challenges remain in accurately correcting for the effects of iodinated contrast agents, particularly in hypovascular or necrotic metastases.

In the present study, TNC imaging was not included as part of the protocol, and VNC imaging was not assessed. Future investigations comparing the diagnostic performance of TNC imaging, VNC imaging, and contrast-enhanced CT parameters in differentiating hypodense liver lesions could provide valuable insights. Understanding the accuracy of VNC imaging in this setting could help to define its role as a potential replacement for TNC imaging, especially in high-risk CRC populations requiring frequent follow-up imaging.

This study’s retrospective nature and single-center design introduce inherent biases, and our results are specific to a single dual-source DECT system. The results are specifically applicable to the contrast administration protocol and acquisition parameters used in our institution and may not be generalizable to other protocols. Furthermore, no direct histopathologic correlation was performed in cases where overlapping Zeff values between metastases and cysts were observed. Further studies integrating histological features with Zeff measurements will be needed to clarify these findings more comprehensively. We also acknowledge that histological confirmation represents the only true gold standard for the definition and discrimination of lesions. However, in cases where biopsy was not available or feasible, contrast-enhanced MRI was used as a secondary reference standard. MRI is a widely accepted, non-invasive diagnostic tool that plays a fundamental role in the evaluation of liver lesions. Its diagnostic performance has been extensively validated in the literature. Meta-analyses have demonstrated high sensitivity and specificity, reported as 0.90 (95% CI: 0.81–0.95) and 0.88 (95% CI: 0.80–0.92), respectively, confirming its reliability in identifying colorectal liver metastases [33]. Given these robust diagnostic capabilities, MRI provides a clinically justified alternative when histological confirmation is not obtainable. Additionally, the exclusion of other hypodense lesions limits the generalizability of our results. Future multicenter studies encompassing broader lesion types and utilizing diverse DECT platforms are essential to validate and extend these observations.

## 5. Conclusions

In conclusion, while conventional CT remains a cornerstone in the evaluation of hypodense liver lesions in CRC patients, with robust diagnostic accuracy, FF was demonstrated to outperform any other parameter in distinguishing between liver cysts and metastasis, improving diagnostic performance. While DECT-derived Zeff maps did not add value in this study, the ongoing evolution of DECT technology may yet reveal new applications. Our findings reinforce the importance of contrast-enhanced CT in routine oncologic imaging, paving the way for more efficient and reliable patient care.

## Figures and Tables

**Table 1:** Demographic characteristics of study population.

	Population	Age	Metastases	Cysts
Men	71	65 ± 12	79	46
Women	49	65 ± 13	68	30
Total	120	65 ± 12	147	76

**Table 2:** Quantitative values of CT measurements, fat fraction, Zeff, and IC in CRC liver metastases and cysts.

	Cysts	Metastases	*p*-Value
Attenuation (HU)	11.9 [9.4–16.2]	34.3 [27.8–42.5]	<0.0001
Fat Fraction (%)	7.2 [4.7–10.9]	25.3 [20.7–27.6]	<0.0001
Atomic Number (Z_eff_)	7.7 [7.5–8.1]	7.7 [7.6–7.9]	0.781
Iodine Concentration (mg/mL)	0.7 [0.5–1]	1.6 [1.3–1.8]	<0.0001

**Table 3:** Intra- and inter-observer agreement assessed using intraclass correlation coefficient (ICC).

	Intra-Reader Agreement Observer 1	Intra-Reader Agreement Observer 2	Inter-Reader Agreement
Attenuation	0.94 (0.93–0.96)	0.93 (0.91–0.95)	0.93 (0.91–0.94)
Fat Fraction	0.86 (0.82–0.89)	0.86 (0.82–0.89)	0.85 (0.81–0.88)
Z-eff	0.88 (0.85–0.91)	0.89 (0.86–0.92)	0.90 (0.87–0.92)
Iodine Concentration	0.87 (0.83–0.90)	0.88 (0.85–0.91)	0.88 (0.85–0.91)

**Table 4:** Diagnostic performance of CT values (HU), fat fraction (FF), atomic number (Zeff), and iodine concentration (IC) in distinguishing between CRC liver metastases and cysts.

	AUC (95% CI)	Cut-Off	Sensitivity (95% CI)	Specificity (95% CI)	Comparison (*p*-Value)		
Attenuation (HU)	0.90 (0.85–0.94)	>19.8	91.8 (86.4–95.7)	75 (63.7–84.2)	FF 0.0074	Zeff <0.0001	IC 0.6419
Fat Fraction (%)	0.97 (0.94–0.99)	>15.9	91.8 (86.2–95.7)	98.7 (92.9–100)	HU 0.0074	Zeff <0.0001	IC 0.0018
Atomic Number (Z_eff_)	0.51 (0.44–0.58)	>7.51	76.2 (68.5–82.8)	34.2 (23.7–46.0)	HU <0.0001	FF <0.0001	IC <0.0001
Iodine Concentration (mg/mL)	0.91 (0.87–0.95)	>1.2	76.2 (68.5–82.8)	90.8 (81.9–96.2)	HU 0.6419	FF 0.0018	Zeff <0.0001

## Data Availability

The data presented in this study are available on request from the corresponding author.

## Abbreviations

The following abbreviations are used in this manuscript:

AUC	area under the curve
CRC	colorectal cancer
CT	computed tomography
DECT	dual-energy computed tomography
FF	fat fraction
GRE	gradient echo
HASTE	half-Fourier acquisition single shot turbo spin echo
IC	iodine concentration
IQR	interquartile range
MRI	magnetic resonance
PACS	picture archiving and communication system
ROC	receiver-operating characteristic curve
ROI	region of interest
SD	standard deviation
TNC	true non-contrast
VIBE	volumetric interpolated breath-hold examination
VNC	virtual non-contrast
Zeff	atomic number
